# Influenza virus-flow from insects to humans as causative for influenza seasonality

**DOI:** 10.1186/s13062-020-00272-5

**Published:** 2020-10-09

**Authors:** Albrecht Pfäfflin

**Affiliations:** Labor Prof. Dr. G. Enders MVZ GbR, Zentrallabor, Hirschlandstr. 97, Esslingen, 73730 Germany

**Keywords:** Influenza, Insects, Seasonality, Virus, Evolution, Compartmentalization

## Abstract

Virus biomass outweighs human biomass, and insects biomass outweighs human biomass. Insects are regularly habited by viruses as well as humans, humans are further inhabited via insects. A model of viral flow is described and specified to explain influenza virus seasonality, which, in temperate climate, usually evolves when insects have mostly disappeared. With this hypothesis a coherent description of regular seasonal influenza and other seasonal respiratory virus infections in temperate climates is possible. The incidence of influenza under different circumstances e.g. temperature, humidity, or tropical conditions and different aspects like synchronicity of infections or in respect to evolutionary conditions do sustain this hypothesis if the behaviour of insects is considered.

## Background

Seasonality of influenza is not understood. The complex transmission behaviour of influenza is enigmatic [1]. The clock-like consistency of the winter incidence peaks of influenza virus in temperate climatic regions represents a strong example of seasonality in infectious disease [2]. Approaching this phenomenon via biomass of involved organisms, a flow may explain this phenomena.

Influenza is considered a zoonosis. The reservoir of influenza virus are aquatic birds [3] which have usually enteral infections often without clinical signs. Influenza virus crosses species barriers from time to time and persists in the species (e.g. humans, dogs, horses) for a certain time in a seasonal manner, and is then eventually lost. Crossing species barriers leads eventually to pandemics [4] which are followed by seasonal epidemics. The question and focus of this paper is laid on the consecutive regular epidemics. When influenza virus has reached humans and persists there, it disappears during off-season but re-emerges regularly. The question is, where does the virus persist during the off-season time of more than 6 months? Several molecular studies suggesting a lack of influenza virus persistence in the off-season in temperate areas [5, 6]. The reintroduction of influenza virus is thought to involve the importation from a locality either in the alternate hemisphere where the influenza season is current, or from the tropics where low levels of virus may circulate year-round, particularly the densely populated regions of East and South-East Asia [7, 8]. However, in this paper, an alternative view is proposed.

### Hypothesis for influenza seasonality via viral-flow

In this model, insects serve as a buffer for influenza virus. If insects are intact, they enclose virus particles preventing these particles to reach humans. If insects deteriorate, virus particles are set free, and humans are infected. So, according to season and insect biomass, a flow of virus particles is ping-ponged between humans and insects. Seasonality of influenza is explainable using this insect-compartment model in temperate climate conditions. If this scenario is truth, it may be speculated, that virus is retransmitted from humans to insects (where virus eventually proliferates), and the circle would be closed.

In this model, insects serve as a connecting transmitter from and to humans to coordinate regular epidemics. They connect birds to humans or other animals and come close to these species. Insects serve as a reservoir for viruses and are able to transport different viral species. When the cold season starts, insects may have, because of the flyaway of many birds, eventually an interest to come in proximity to humans. Humans provide them warmness and nutrition, otherwise this proximity fosters viral transmission from insects to humans.

### Sustaining arguments for this hypothesis

It is not possible, to proof this hypothesis, but it is possible to describe circumstances which fit to this hypothesis. Such circumstances are discussed in the following.

#### Biomass composition

Biomass composition of the earth [9] is such: Half of animals are arthropods. There are 17fold more arthropod biomass compared to humans. There is a 3fold biomass of virus compared to humans. Insects are living closer to humans as expected [10]. Arthropods and viruses predate humans, and held and hold manifold interactions. Viruses predate any life [11]. Only because of these mass relations, an interaction of viruses with arthropods seems plausible and is found in numerous examples e.g. Zika-virus and *Aedes albopictus*. Indeed, insects are able to transmit influenza virus, experimentally proven at least in birds [12], and, insects e.g. musca indeed act as vectors [13, 14] at least for avian influenza. Life of vertebrates does not occur without insects even under current conditions. Some centuries ago, the interactions of humans with insects were, compared to now, because of a more simple life style, surely more intense. So, an exchange of insect contaminants is unavoidable.

In analogy, transmission of virus from plants to plants takes place mostly via insects [15]. Many details of these interactions have been elucidated revealing a high-level of complexity between plant-viruses and insects. In this context, it is astonishing that the transmission of human influenza virus via insects is yet neglected.

#### Epidemiological and clinical hints

An argument for insects as an influenza vector are their presence during off-season of influenza and vice versa. Isolated insect-free regions without virus do not show seasonality of influenza [16]. Human immune response is not obligatory discriminative between influenza and insect structures indicating eventually that insect and influenza structures are linked [17]. A synergy between insects and influenza may indicate an ancient coupling between these organisms [18]. An ecological niche that is currently underrepresented in global surveillance efforts was postulated, namely the rare influenza subtype H14 was found only after several decades [19]. Such an ecological niche in the environment would perfectly fit to insects. These arguments have been at least partly discussed as an insect-compartment model contributory to explain seasonality of influenza [20].

#### Environmental conditions

Influenza epidemics occur usually in synchronicity [21]. Seasons in temperate climates begin when temperatures are permanently low. Direct human to human transmission does not cause synchronicity because of a lag-time during the incubation period. Synchronicity is to be triggered to happen regularly, therefore natural processes may be responsible for. Insects emerge and cease in a synchronized manner. Humans may act, in this context, as a dead-end host for influenza according to a source-sink model for the ecology of influenza [22]. Synchronicity of influenza may be triggered via environmental conditions. Temperature and absolute humidity are revealed to be important for transmission [23] as well as low temperature [24]. Cold and dry air facilitates virus stability [25]. However, such observations do not explain why influenza disappears and where it stays in the meantime, but such factors will influence survival of insects as well.

Global weather conditions are connected to pandemic influenza [26]. Associations of global weather conditions to the dynamics of seasonal influenza are found regularly, but the biological mechanism between climate variations and influenza epidemics is dubious [27]. Insects might be this biological mechanism. Weather conditions imperatively will affect the biomass of insects and migration birds. General weather phenomenon e.g. El Nino Southern Oscillation probably may lead also to an increase or decrease of insect biomass providing a biological reason for observed associations [28] viewed from the viral-flow model.

Seasonality of influenza in the tropics is also not well understood because accepted influence factors like temperature or absolute humidity show weak or absent associations in tropical countries. Epidemics occur there during the rainy season [27]. Eventually, insect biomass is different during and out of rainy season fitting to the model proposed here.

### Consequences

Accepting this approach may imply eventually new proceedings for prevention of influenza e.g. distribution of insects or insect particles to neutralize virus. A kind of therapeutic use of insect particles [29] was already described.

## Discussion and concluding remarks

Here, an viral-flow model contributory to explain seasonality of influenza is applied to elucidate questions and circumstances concerning influenza infections like synchronicity, environmental factors. With this approach, an insect-compartment relevant for the transmission of influenza is not proven, and other transmission routes e.g. direct transmission or transmission via aerosols are active as well, but this approach provides answers to question where influenza virus persists between epidemics, and how it is reintroduced regularly. Of note, many other seasonal viruses show an enigmatic seasonal behaviour like parainfluenza, respiratory syncytial, human metapneumovirus and others. They may act in a similar way as described here.

The human-centred view on virus transmission does not allow recognizing a viral flow. This human-centred view is the perception that viral infections are rare and viruses proliferate preferentially or exclusively in humans. Viruses are invisible and difficult to detect. Therefore, the notification of viral-flow is an abstraction and unusual as a concept, but, from an evolutionary point-of-view, at least debatable.

## Data Availability

Not applicable.
